# Recurrent Trigeminal Neuralgia: A Case Series and a Review of the Literature

**DOI:** 10.7759/cureus.22548

**Published:** 2022-02-23

**Authors:** Harsha Vardhan, Sushmitha S, Nagammai N, Saraswathi K

**Affiliations:** 1 Oral Medicine and Radiology, Meenakshi Ammal Dental College, Chennai, IND; 2 Faculty of Dentistry, Oral Medicine and Radiology, Sri Ramachandra Institute of Higher Education and Research, Chennai, IND

**Keywords:** microvascular decompression, mr imaging, recurrence, neuropathic pain, trigeminal neuralgia

## Abstract

Trigeminal neuralgia is a peripheral neuropathy characterized by intermittent episodes of severe facial pain originating in the sensory nucleus of the trigeminal nerve. The most commonly involved area is the mandibular division with a higher prevalence on the right side. Advances in the field of MRI have played an important role in its diagnosis, especially in presurgical assessment, to probe into any secondary causes of nerve compression and/or neurovascular conflict. The condition is primarily managed medically, although many patients may require surgical or radiotherapeutic intervention. A recurrence rate ranging from 6 to 41% has been postulated. Reasons for recurrence are mainly attributed to improper operative techniques, dislocation of the Teflon implant, or granuloma formation. MRI serves as a powerful tool in the segmental evaluation of the trigeminal nerve. A proper diagnosis with a structured treatment protocol is critical for managing such cases of trigeminal neuralgia. In this report, we present a series of two cases of recurrent trigeminal neuralgia.

## Introduction

Trigeminal neuralgia is also referred to as Tic douloureux, Fothergill’s disease, and suicide disease. It is characterized by a sudden, unilateral, recurrent lancinating pain that radiates through one or more branches of the fifth cranial nerve [1]. According to the International Headache Society, trigeminal neuralgia is a condition defined by the occurrence of attacks lasting between one second and two minutes that affect several subdivisions of the trigeminal nerve with the affected individual experiencing an intense, sharp, superficial, or stabbing pain precipitated by specific triggers or actions. The attacks are characteristic for each individual patient with no clinically evident neurological deficit [2]. With the progression of the disease course, patients may have difficulty in performing their day-to-day activities as they will be in constant fear of triggering the pain episodes, thereby causing a profound impact on the quality of life [3]. The main etiological factors associated with the condition are direct trauma, nerve compression, or any underlying systemic condition affecting that nerve [4]. It appears to be more prevalent among women and has a higher tendency to occur on the right side of the head.

The various treatment modalities of trigeminal neuralgia include pharmacological management, rhizotomy, laser therapy, balloon compression, surgical intervention, and stereotactic radiosurgery. Among the different treatment methods, the most common treatment of choice is surgical management, but a major drawback is the condition's recurrence rate. Many literature reviews cite a recurrence rate ranging from 6 to 41% [5]. Currently, the preferred surgical treatment for primary trigeminal neuralgia is microvascular decompression (MVD) [6]. It was popularized by Jannetta in the year 1980. The operation aims to pad the contact of an irritating arterial vessel with the trigeminal nerve by using polytetrafluoroethylene. In a study by Neto et al. among patients who underwent surgical management for trigeminal neuralgia, 64% experienced asymptomatic pain relief without pain medications for 10 years, while 4% experienced only partial pain relief [7]. After a time period of five years, the recurrence rate was 2%, and it was 1% after 10 years. The post-management recurrence of the condition is mainly attributed to inappropriate operative techniques.

High-resolution MRI is reported to have excellent sensitivity and specificity in the pre and postoperative imaging of primary, secondary, and postoperative trigeminal neuralgia and in cases of recurrent trigeminal neuralgia. Against this background, this article presents a series of two cases and engages in a detailed review of the etiology and imaging characteristics of recurrent trigeminal neuralgia.

## Case presentation

Case 1

**Figure 1:**
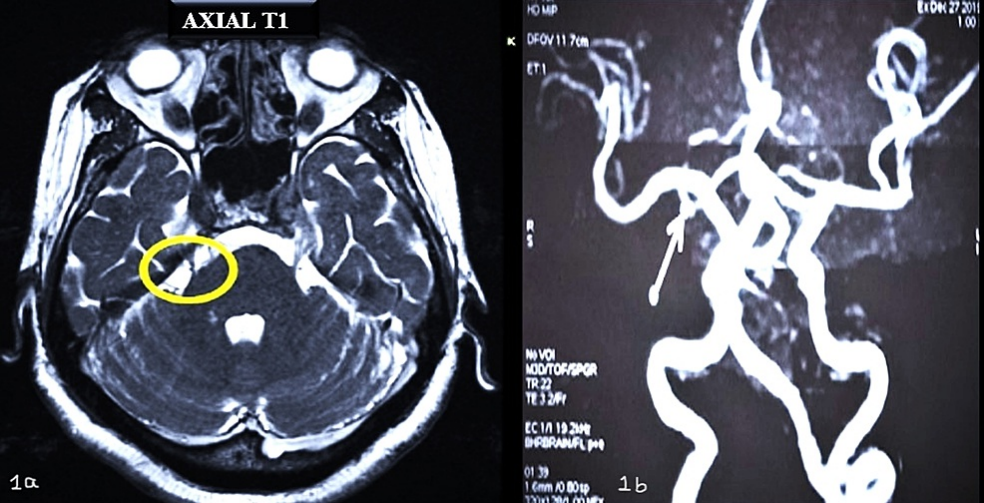
MRI (1a) and MRA (1b) findings show a vascular loop from the right superior cerebellar artery that was abutting and indenting the right trigeminal nerve at the cisternal segment and root entry zone MRI: magnetic resonance imaging; MRA: magnetic resonance angiography

A 64-year-old female patient reported to the department with the chief complaint of pain on the right side of the face for the past week. History revealed pain in relation to her right upper tooth region for a duration of two weeks for which she had undergone restoration a week prior. Following the restoration, she experienced five intermittent episodes of electric shock-like pain in relation to the right middle-third of the face, each lasting for 10-15 seconds. The pain was triggered by washing the face and exposure to a cool breeze. Her medical history revealed surgical management with MVD for the same complaint four years ago. Post-surgery, she had been on regular medication with pregabalin tablet 75 mg but discontinued it on her own two months prior to the presentation. Correlating the patient’s previous medical history and clinical examination, a provisional diagnosis of recurrent trigeminal neuralgia of the right side was made. The patient subsequently underwent MRI and MRA examinations, which revealed a vascular loop from the right superior cerebellar artery that was seen abutting and indenting the right trigeminal nerve at the cisternal segment and nerve root entry zone, suggestive of type I neurovascular conflict (Figures 1a, 1b). The patient was managed with carbamazepine 200 mg in the morning and pregabalin 75 mg at night. Post-medication, the patient experienced significant relief from the intermittent pain episodes followed by complete relief within a week.

Case 2

**Figure 2:**
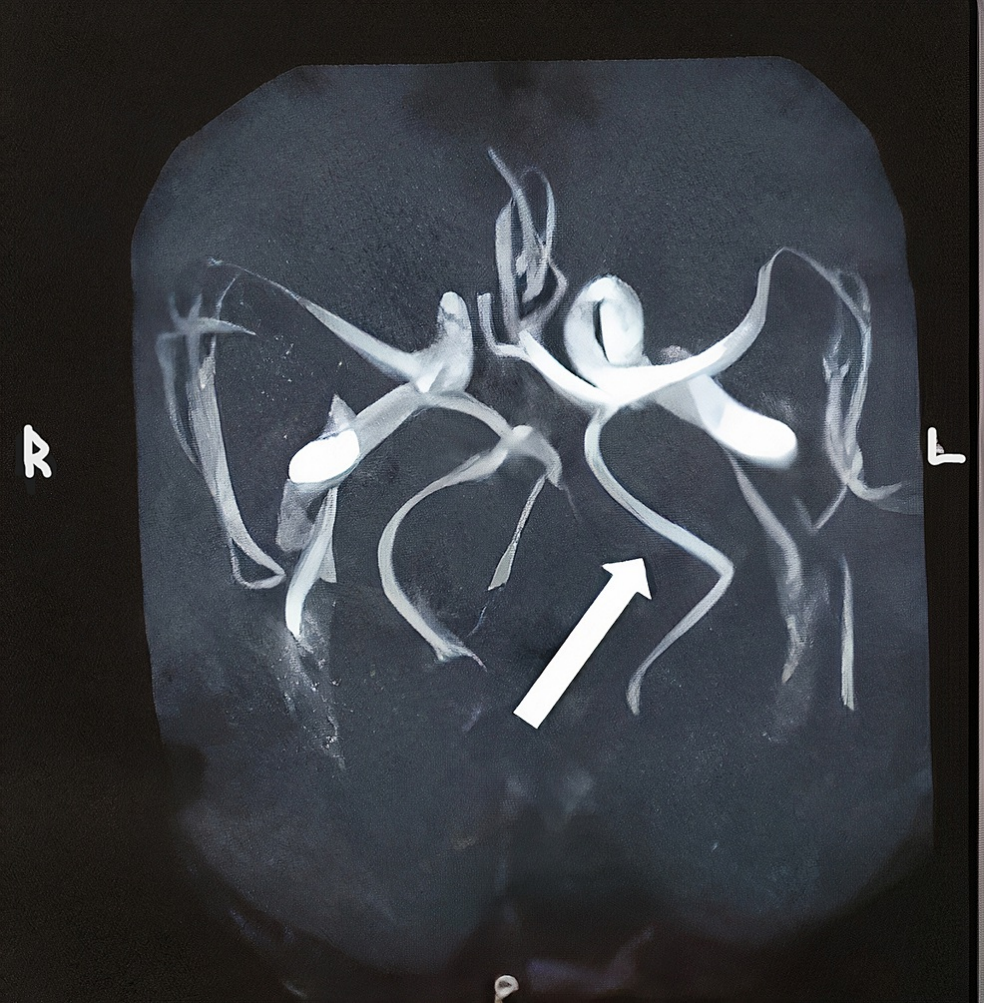
MRA imaging shows age-related changes with hypoplastic V4 segment of left vertebral artery with poor blood flow and the fetal origin of left posterior cerebral artery MRA: magnetic resonance angiography

A 66-year-old male patient reported to the department with the chief complaint of pain on the left side of the face for the past 15 days. History of presenting illness revealed pain in the left cheek region close to the left ala of the nose and forehead region. The pain was intermittent, electric shock-like, and each episode lasted for 10-20 seconds. The pain was triggered by touching, washing the face, sneezing, and also by exposure to the breeze. The patient had been experiencing about two to three episodes of pain in a day, but the episodes and their intensity had increased over the past three days. No pain was felt during sleeping hours. The patient also reported experiencing a similar kind of pain in the same region and in a similar fashion 10 months ago, which had led to a diagnosis of trigeminal neuralgia. He had been managed with carbamazepine 200 mg and amitriptyline hydrochloride 25 mg for a period of two months, following which he discontinued the therapy without any medical intervention. Similar episodes of pain had begun just 15 days ago, which led him to seek medical advice. Correlating the past medical history and present clinical status, the patient was diagnosed with recurrent trigeminal neuralgia of the left maxillary division (Figure 2). The patient underwent an MRA, which revealed age-related changes in the hypoplastic V4 segment of the left vertebral artery with poor blood flow (Figure 2). The patient was subsequently managed with carbamazepine 200 mg twice a day and amitriptyline hydrochloride 75 mg once a day for six months. He is currently undergoing regular periodic follow-ups.

## Discussion

Trigeminal neuralgia has been described by the Classification Committee of the International Association for the Study of Headache as “a painful unilateral affliction of the face, characterized by brief electric shock-like (lancinating) pain restricted to one or more divisions of the trigeminal nerve. Washing, shaving, smoking, talking, and brushing the teeth are common trivial triggers that may produce pain, but it can also happen spontaneously. The pain is abrupt in onset and may remit for varying periods” [8]. A proper diagnostic workup of trigeminal neuralgia is very much necessary to distinguish it from classic idiopathic neuralgia and secondary symptomatic neuralgia. The primary or classic idiopathic trigeminal neuralgia does not have any underlying cause whereas symptomatic trigeminal neuralgia is associated with an underlying structural lesion or multiple sclerosis. The overall prevalence of trigeminal neuralgia is 5.9 per 1,00,000 among women and 3.4 per 1,00,000 in men [1]. The incidence tends to be slightly higher in women, with the right side of the face more predominantly involved than the left side, which is mainly due to the smaller size of the foramen ovale and rotundum.

Various theories have been proposed regarding the pathogenesis of the trigeminal nerve, such as the neurovascular compression theory and trigeminal convergence theory [9]. Neurovascular compression theory attributes the condition to arteriovenous malformation or vestibular schwannomas or meningioma, which may compress the root entry zone of the trigeminal nerve and cause instability with demyelination. According to the trigeminal convergence theory, the condition is caused by continuous or recurrent nociceptive inputs from the head and neck that converge to the spinal nucleus, which causes the release of neurotransmitters that in turn excites the third-order neuron and converge into the thalamus.

Pain is often felt in the areas of the nerve distribution zones such as ophthalmic (V1), maxillary (V2), and mandibular (V3) divisions. The mandibular division is most commonly involved (55%) followed by the maxillary division (39%), while the ophthalmic division is the least involved (6%). A combination of V2 and V3 occurs in 9%, and in 2% of cases, all the three divisions are involved. This could explain the pronounced symptoms in V3 and V2 compared to the V1 branch [10-11].

MRI could be beneficial for mapping the entire course of the trigeminal nerve right from its origin in the brain stem nuclei to its peripheral branches. Its major advantages include the high resolution, increased number of acquisitions, and the imaging of the particular segment of the nerve. The trigeminal nerve could be imaged at five segments: the brain stem, cisternal angle, Meckel's cave, cavernous sinus, and peripheral segments. In the brain stem, the segment houses the central component of the trigeminal nerve comprising both motor and sensory nuclei. The anomalies affecting the brain stem are multiple sclerosis, arteriovenous malformation, and infarcts. The cisternal angle segment and Meckel's cave are imaged together. It entails the emergence of sensory and motor roots traversing into the middle cranial fossa by piercing the dura and entering Meckel's cave. Three major branches emerge from the sensory nerve root: the ophthalmic V1, maxillary V2, and mandibular V3. The motor root passes beneath the ganglion and the pathologies affecting these segments include schwannoma, lipoma, meningioma, epidermoid, lymphoma, and acoustic schwannoma. The cavernous sinus segment aids in imaging both ophthalmic and maxillary divisions along with their associated diseases including trigeminal schwannoma, cavernous-carotid aneurysm, and Tolosa-Hunt syndrome. The peripheral segment imaging involves a detailed view of the sub-branches of all the three major branches [12].

MRA aids in ruling out the neurovascular conflict etiology of trigeminal neuralgia. It helps to generate images of arteries in order to evaluate them for stenosis, occlusions, and aneurysms [13]. In 86.6% of cases, the superior cerebellar artery, a branch of the basilar artery from the circle of Willis, causes compression on the root entry zone of the trigeminal nerve.

There has been a significant increase in the utilization of different magnetic resonance techniques, with recent introductions including voxel-based morphometry [14], diffusion tensor imaging, three-dimensional time-of-flight MRA, and fluid-attenuated inversion recovery sequences [15]. These advances in MRI have been playing an important role in the diagnostic setting, especially in the presurgical evaluation [16]. Moisset et al. [17] have shown that the trigger of painful stimuli was associated with significantly increased signal activity in the spinal trigeminal nucleus, thalamus, primary and secondary somatosensory cortices, anterior cingulate cortex, insula, premotor/motor cortex, prefrontal areas, putamen, hippocampus, and brainstem. Pertaining to the non-painful stimulation of the trigger zone, an increased activation of signal intensity was only observed in three structures: spinal trigeminal nucleus, brainstem, and anterior cingulate cortex. After surgical management, the activation of the operated side was confined to primary and secondary somatosensory cortices. A reduction in the gray matter volume in trigeminal neuralgia cases was also noted in the voxel-based morphometry MRI sequence. Liu et al. found that the affected side showed significantly decreased fractional anisotropy and increased radial diffusivity, suggesting that demyelination without significant axonal injury is an important factor in trigeminal neuralgia pathogenesis [15]. The hypothesis in our second case is attributed to age-related changes in the brain and poor vascular supply that could possibly lead to the diagnosis of recurrent trigeminal neuralgia.

Phenytoin was the first drug used for trigeminal neuralgia with reported positive effects. However, according to the recent European Federation of Neurological Societies (EFNS) guidelines, two drugs are considered as first-line therapy in trigeminal neuralgia: carbamazepine (CBZ; 200-1,200 mg/day) and oxcarbamazepine (OXC; 600-1,800 mg/day) [18]. Various surgical approaches have been proposed for the treatment of drug-resistant trigeminal neuralgia. MVD is frequently performed with the objective to resolve the primary neurovascular conflict between an abnormal vessel and the trigeminal nerve [19]. It has been suggested by various longitudinal studies that this surgical modality might provide short- and long-term pain relief in more than 90% of patients. However, one-third of patients have experienced recurrences within five years of undergoing MVD. Most often, the recurrences were caused by compression by an artery, vein, or simply the Teflon pad, whereas 30% occurred without any apparent cause [20-21]. In our first case discussed here, the patient developed recurrence within four years after management with MVD.

Other modalities of management are through percutaneous invasive procedures, a method involving a trans foramen ovale approach to the retrogasserian portion of the trigeminal nerve, and Gamma Knife radiosurgery (GKRS) aiming at damaging the trigeminal nerve root with a high and concentrated dose of radiation. Percutaneous balloon compression provides immediate postoperative pain relief ranging from 80 to 90% and a pain-free period without medication that ranges from two to three years [22-23]. Glycerol rhizotomy involves the injection of glycerol in the trigeminal cistern segment, providing pain relief in patients due to demyelination and axonal fragmentation [24]. GKRS has been used as a treatment modality for patients with concurrent medical illnesses incompatible to be treated with MVD or patients who do not opt for invasive surgery. The radiosurgery is carried out with a radiation dose protocol ranging from 70 to 100 Gy at the root entry zone of the trigeminal nerve in an effort to alleviate pain [25-26]. By assessing patients' responses to non-invasive cortical stimulation first, repetitive transcranial magnetic stimulation (rTMS) allows for assessing whether they will respond to epidural cortical stimulation in case of trigeminal neuropathic pain; 24 patients participated in a study where they received rTMS twice daily for five days at 20 Hz. Within two weeks, pain ratings decreased by approximately 45% [27].

The most frequent causes of recurrence in trigeminal neuralgia are thought to be incomplete decompression, recompression of the root exit zone by migration of an inserted prosthesis, adhesion or fibrosis between the offending artery and the root exit zone because of an inappropriately inserted prosthesis, and retitled axis of the nerve by postoperative arachnoid adhesion [28]. Aside from pharmacological management, recurrent trigeminal neuralgia cases may be treated via repeat MVDs, performing percutaneous glycerol or radiofrequency rhizotomy, stereotactic radiosurgery, or performing percutaneous balloon compression [29]. Cheng et al. have reported an 80% rate of pain relief with repeat MVD within a mean period of two years, and a further 7% of patients experienced partial pain relief. But the complications were higher in repeat MVD, such as hearing loss in 5%, wound infection in 2.5%, and cerebrospinal fluid leak in 2.5% of cases. According to Raygor et al. [30], repeat stereotactic radiosurgery was only effective in 33.3% of the cases of persistent trigeminal neuralgia, compared to 80% with redo MVD procedures. In patients reporting recurrent symptoms, MRI is mandatory to determine where the padding is, which artery is offending, as well as the deformity of the nerves [31].

## Conclusions

Even though trigeminal neuralgia is a relatively rare disease, it is often associated with debilitating pain and disability. Various other neurological diseases may mimic its symptoms, and hence appropriate diagnosis is necessary prior to the initiation of its management. There are many factors that trigger a recurrence of trigeminal neuralgia after a successful initial MVD, including nerve deformation from adhesions and continual irritation by the offending vessel. MRI with its various advanced sequences serves as a powerful imaging tool in the segmental evaluation of the trigeminal nerve. Thus, a proper diagnosis with a structured treatment protocol is imperative in managing recurrent trigeminal neuralgia cases.

## References

[1] Zakrzewska JM, Linskey ME (2014). Trigeminal neuralgia. BMJ.

[2] Headache Classification Subcommittee of the International Headache Society (2004). The International Classification of Headache Disorders: 2nd Edition. Cephalalgia.

[3] Moore D, Chong MS, Shetty A, Zakrzewska JM (2019). A systematic review of rescue analgesic strategies in acute exacerbations of primary trigeminal neuralgia. Br J Anaesth.

[4] Maarbjerg S, Gozalov A, Olesen J, Bendtsen L (2014). Trigeminal neuralgia--a prospective systematic study of clinical characteristics in 158 patients. Headache.

[5] Rabinovich A, Fang J, Scrivani SJ (2005). Diagnosis and management of trigeminal neuralgia. Columbia Dent Rev.

[6] Chen ZW, Ma JB, Xie KY, Huang B, Yao M, Fei Y, Zhang L (2018). A study of the relations of foramen rotundum structure direction and the approach of percutaneous puncturing of radiofrequency thermocoagulation for treating V2 of primary trigeminal neuralgia (Article in Chinese). Zhonghua Yi Xue Za Zhi.

[7] Neto HS, Camilli JA, Marques MJ (2005). Trigeminal neuralgia is caused by maxillary and mandibular nerve entrapment: greater incidence of right-sided facial symptoms is due to the foramen rotundum and foramen ovale being narrower on the right side of the cranium. Med Hypotheses.

[8] Headache Classification Committee of the International Headache Society (1988). Classification and diagnostic criteria for headache disorders, cranial neuralgias and facial pain. Headache Classification Committee of the International Headache Society. Cephalalgia.

[9] Sabalys G, Juodzbalys G, Wang HL (2013). Aetiology and pathogenesis of trigeminal neuralgia: a comprehensive review. J Oral Maxillofac Res.

[10] Casey KF (2005). Role of patient history and physical examination in the diagnosis of trigeminal neuralgia. Neurosurg Focus.

[11] Bangash TH (2011). Trigeminal neuralgia: frequency of occurrence in different nerve branches. Anesth Pain Med.

[12] Becker M, Kohler R, Vargas MI, Viallon M, Delavelle J (2008). Pathology of the trigeminal nerve. Neuroimaging Clin N Am.

[13] Korogi Y, Nagahiro S, Du C (1995). Evaluation of vascular compression in trigeminal neuralgia by 3D time-of-flight MRA. J Comput Assist Tomogr.

[14] Obermann M, Rodriguez-Raecke R, Naegel S (2013). Gray matter volume reduction reflects chronic pain in trigeminal neuralgia. Neuroimage.

[15] Liu Y, Li J, Butzkueven H (2013). Microstructural abnormalities in the trigeminal nerves of patients with trigeminal neuralgia revealed by multiple diffusion metrics. Eur J Radiol.

[16] Zeng Q, Zhou Q, Liu Z, Li C, Ni S, Xue F (2013). Preoperative detection of the neurovascular relationship in trigeminal neuralgia using three-dimensional fast imaging employing steady-state acquisition (FIESTA) and magnetic resonance angiography (MRA). J Clin Neurosci.

[17] Moisset X, Villain N, Ducreux D (2011). Functional brain imaging of trigeminal neuralgia. Eur J Pain.

[18] Cruccu G, Sommer C, Anand P (2010). EFNS guidelines on neuropathic pain assessment: revised 2009. Eur J Neurol.

[19] Jannetta PJ (1967). Arterial compression of the trigeminal nerve at the pons in patients with trigeminal neuralgia. J Neurosurg.

[20] Cong L, Zhihua C, Zhilin G, Huoniu OY (2019). Effects of microvascular decompression plus longitudinal nerve sectioning on recurrent trigeminal neuralgia and investigations of postoperative recurrence causes. Turk Neurosurg.

[21] Raygor KP, Wang DD, Ward MM, Barbaro NM, Chang EF (2018). Long-term pain outcomes for recurrent idiopathic trigeminal neuralgia after stereotactic radiosurgery: a prospective comparison of first-time microvascular decompression and repeat stereotactic radiosurgery. J Neurosurg.

[22] Brown JA, McDaniel MD, Weaver MT (1993). Percutaneous trigeminal nerve compression for treatment of trigeminal neuralgia: results in 50 patients. Neurosurgery.

[23] Jafree DJ, Zakrzewska JM (2019). Long-term pain relief at five years after medical, repeat surgical procedures or no management for recurrence of trigeminal neuralgia after microvascular decompression: analysis of a historical cohort. Br J Neurosurg.

[24] Kondziolka D, Lunsford LD (2005). Percutaneous retrogasserian glycerol rhizotomy for trigeminal neuralgia: technique and expectations. Neurosurg Focus.

[25] Young RF, Vermeulen SS, Grimm P, Blasko J, Posewitz A (1997). Gamma Knife radiosurgery for treatment of trigeminal neuralgia: idiopathic and tumor related. Neurology.

[26] Montano N, Conforti G, Di Bonaventura R, Meglio M, Fernandez E, Papacci F (2015). Advances in diagnosis and treatment of trigeminal neuralgia. Ther Clin Risk Manag.

[27] Khedr EM, Kotb H, Kamel NF, Ahmed MA, Sadek R, Rothwell JC (2005). Longlasting antalgic effects of daily sessions of repetitive transcranial magnetic stimulation in central and peripheral neuropathic pain. J Neurol Neurosurg Psychiatry.

[28] Inoue H, Kondo A, Shimano H, Yasuda S (2013). Recurrent trigeminal neuralgia at 20 years after surgery: case report. Neurol Med Chir (Tokyo).

[29] Cheng J, Meng J, Lei D, Hui X (2019). Repeat microvascular decompression for patients with persistent or recurrent trigeminal neuralgia: prognostic factors and long-term outcomes. Medicine (Baltimore).

[30] Raygor KP, Lee AT, Nichols N, Wang DD, Ward MM, Barbaro NM, Chang EF (2020). Long-term pain outcomes in elderly patients with trigeminal neuralgia: comparison of first-time microvascular decompression and stereotactic radiosurgery. Neurosurg Focus.

[31] Aljuboori Z, Nauta HJ (2019). Multiple recurrences of trigeminal neuralgia caused by deformation of the trigeminal nerve. Cureus.

